# T cell–derived tumor necrosis factor induces cytotoxicity by activating RIPK1-dependent target cell death

**DOI:** 10.1172/jci.insight.148643

**Published:** 2021-12-22

**Authors:** Nicholas Chun, Rosalind L. Ang, Mark Chan, Robert L. Fairchild, William M. Baldwin, Julian K. Horwitz, Jesse D. Gelles, Jerry Edward Chipuk, Michelle A. Kelliher, Vasile I. Pavlov, Yansui Li, Dirk Homann, Peter S. Heeger, Adrian T. Ting

**Affiliations:** 1 Department of Medicine and Translational Transplant Research Center and; 2 Precision Immunology Institute, Icahn School of Medicine at Mount Sinai, New York, New York, USA.; 3 Department of Immunology, Mayo Clinic, Rochester, Minnesota, USA.; 4 Department of Inflammation and Immunity, Cleveland Clinic, Cleveland, Ohio, USA.; 5 Graduate School of Biomedical Sciences and; 6 Tisch Cancer Institute and the Department of Oncological Sciences, Icahn School of Medicine at Mount Sinai, New York, New York, USA.; 7 Department of Molecular, Cell and Cancer Biology, University of Massachusetts Medical School, Worcester, Massachusetts, USA.; 8 Diabetes, Obesity & Metabolism Institute, Icahn School of Medicine at Mount Sinai, New York, New York, USA.

**Keywords:** Cell Biology, Immunology, T cells

## Abstract

TNF ligation of TNF receptor 1 (TNFR1) promotes either inflammation and cell survival by (a) inhibiting RIPK1’s death-signaling function and activating NF-**κ**B or (b) causing RIPK1 to associate with the death-inducing signaling complex to initiate apoptosis or necroptosis. The cellular source of TNF that results in RIPK1-dependent cell death remains unclear. To address this, we employed in vitro systems and murine models of T cell–dependent transplant or tumor rejection in which target cell susceptibility to RIPK1-dependent cell death could be genetically altered. We show that TNF released by T cells is necessary and sufficient to activate RIPK1-dependent cell death in target cells and thereby mediate target cell cytolysis independently of T cell frequency. Activation of the RIPK1-dependent cell death program in target cells by T cell–derived TNF accelerates murine cardiac allograft rejection and synergizes with anti-PD1 administration to destroy checkpoint blockade–resistant murine melanoma. Together, the findings uncover a distinct immunological role for TNF released by cytotoxic effector T cells following cognate interactions with their antigenic targets. Manipulating T cell TNF and/or target cell susceptibility to RIPK1-dependent cell death can be exploited to either mitigate or augment T cell–dependent destruction of allografts and malignancies to improve outcomes.

## Introduction

TNF is a pleiotropic cytokine produced by multiple cell types, including monocytes, macrophages, T cells, and endothelial cells (1–3). TNF was originally identified by its ability to kill fibrosarcoma cell lines in vitro and to induce necrosis of implanted tumors in vivo — hence, its name (4, 5). Subsequent studies show that many tumors are resistant to TNF-mediated killing and that systemic TNF administration is not an effective therapy for most human malignancies (6). Later work demonstrating a pathogenic role for TNF in multiple inflammatory disease processes focused attention on the death-independent, proinflammatory functions of the cytokine, and they led to effective neutralization therapies for rheumatoid arthritis and inflammatory bowel disease (7–9).

The discovery of TNF-initiated, regulated cell death — including apoptosis and necroptosis — renewed interest in TNF’s cytotoxic function (10, 11). A large body of research has established that TNF binding to its primary receptor, TNF ligation of TNF receptor 1 (TNFR1), can either promote cell survival or induce cell death. The default response in a majority of cells appears to be cell survival due the presence of multiple checkpoints in the TNFR1 signaling pathway that function to inhibit the cell death machinery (12–14). A key cell death checkpoint is the early ubiquitination of RIPK1 by TRAF2–cIAP1/2 and the LUBAC E3 ligase complexes that catalyze lysine 63 (K63) and linear (M1) poly-ubiquitination, respectively (see schematic in Supplemental Figure 1; supplemental material available online with this article; https://doi.org/10.1172/jci.insight.148643DS1). These modifications serve to inhibit RIPK1 from interacting with the death signaling complex (known as complex II) containing FADD, CASPASE 8, and RIPK3 (15–18). Absence or dysfunction of the E3 ligases and/or upregulation of deubiquitinases permits association of RIPK1 with complex II (15–22). Downstream activation of CASPASE 8 initiates apoptosis, while inhibition of CASPASE 8 facilitates RIPK3 activation, leading to activation of mixed lineage kinase domain-like protein (MLKL), the effector molecule mediating necroptosis (23, 24). Both forms of cell death are dependent on the kinase activity of RIPK1.

While activation of RIPK1-dependent cell death likely evolved in response to certain pathogenic intracellular infections (25–28), accumulating evidence from multiple laboratories revealed that apoptosis and/or necroptosis are pathogenic mediators of several disease processes involving acute tissue injury and repair, including ischemia reperfusion injury and acute kidney injury (29, 30), inflammatory bowel disease (31, 32), cardiovascular disease/stroke (33, 34), and neurodegeneration (35, 36). The source of the TNF that initiates activation of RIPK1-dependent cell death programs under these circumstances has not been definitively determined, although evidence indicates that macrophage production of TNF is crucial (37, 38).

Emerging evidence also implies that RIPK1-dependent programmed cell death modulates outcomes of immunological disease processes, including T cell–mediated rejection of malignancies (14, 39, 40) and organ transplants (41, 42). In support of this contention, previous work by others showed that RIPK3-deficient donor organs undergo rejection with delayed kinetics compared with WT controls (43, 44). Further studies performed in a CD4^+^ T cell–dependent chronic heart rejection model indicated that CD4^+^ T cells can induce RIPK3-dependent endothelial cell death (45) in part by causing mitochondrial dysfunction (46). Despite these clinically relevant observations, the source of TNF that initiates RIPK1-dependent programmed cell death and resultant rejection of tumors or transplants remains obscure. As polyfunctional, cytotoxic CD4^+^ and CD8^+^ T cells can secrete TNF upon cognate interaction with peptide/MHC expressed on target cells (47, 48), we hypothesized that T cell–derived TNF released during cognate interactions with their antigenic target functions as an essential initiator of RIPK1-dependent cell death in target cells and thereby increases the target cell susceptibility to T cell–dependent cytolysis. To test this concept, we employed murine allogeneic transplant systems and syngeneic tumor models in which target tissue destruction requires antigen-reactive T cells and in which target tissue susceptibility to programmed cell death can be manipulated.

## Results

### Recipient TNF and RIPK1-dependent cell death in the graft crucially mediate T cell–dependent cardiac allograft rejection.

To initially extend previously published work by others associating donor RIPK3 deficiency with delayed allograft rejection (43, 44), we employed a distinct model of murine heterotopic heart transplantation in which the donor organ is subjected to prolonged cold ischemic storage (CIS). We have shown previously that this model mimics clinical aspects of deceased donor heart transplantation in humans and results in CTLA4Ig-resistant rejection (49, 50) not observed in standard heart transplant models that lack CIS (43–45).

**Figure 1:**
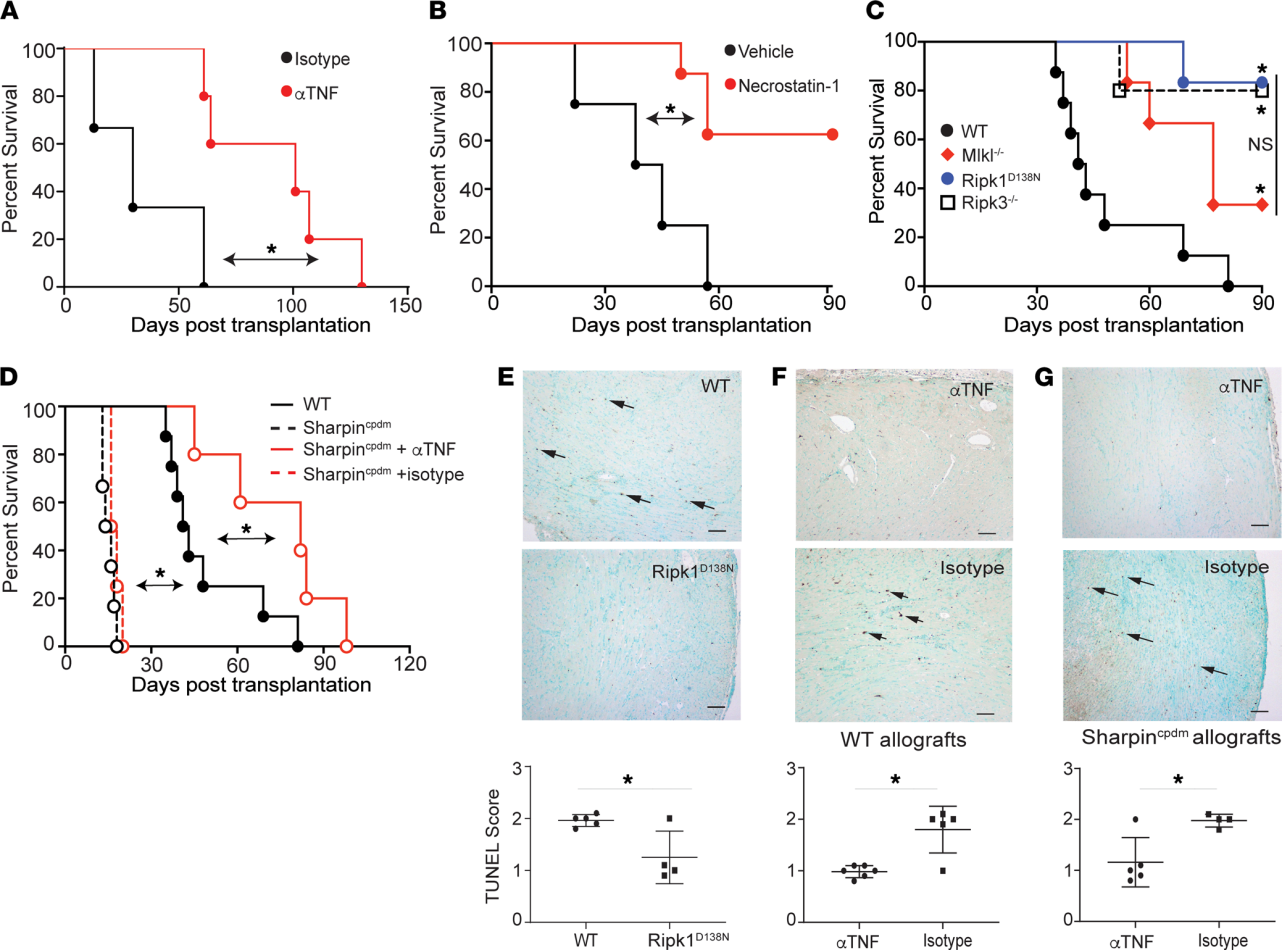
TNF-modulated, RIPK1-dependent cell death mediates graft rejection and intragraft cell death. (**A**–**D**) Kaplan-Meier survival curves for WT or genetically distinct B6 heart grafts subjected to 8 hours (8h) of CIS and transplanted into CTLA4Ig-treated BALB/c recipients. (**A**) WT B6 hearts into recipients treated with anti-TNF mAb (*n* = 5) or isotype IgG (*n* = 3) control on the day of surgery. (**B**) WT B6 hearts into recipients treated with necrostatin-1 (*n* = 8) or vehicle control (*n* = 4) on the day of surgery. (**C**) *Ripk1^D138N^* (*n* = 6), *Mlkl^–/–^* (*n* = 6), *Ripk3^–/–^* (*n* = 5), or WT (*n* = 8) B6 allografts. *P < 0.05 versus WT control. (**D**) WT (same data as shown in **C**) or *Sharpin^cpdm^* B6 allografts transplanted into WT BALB/c recipients ± anti-TNF blocking mAb (αTNF, day 0, then every 5 days for 2 weeks, *n* = 5) or equivalent dosing of IgG isotype control (isotype *n* = 4, *P* < 0.05 versus IgG control). (**E**) Representative TUNEL staining (arrowheads) and quantification of day-7 posttransplant WT and *RIPK1^D138N^* B6 hearts in WT BALB/c recipients (*n* = 4–5). Each dot represents a biological replicate. *P* < 0.05 versus WT control, *t* test. (**F**) Representative TUNEL staining and quantification of WT B6 allografts transplanted as described above into BALB/c hosts treated with anti-TNF mAb or isotype control (*n* = 5–6, *t* test). (**G**) Representative TUNEL staining and quantification of *Sharpin^cpdm^* B6 allografts transplanted as described above into BALB/c hosts treated with anti-TNF mAb or isotype control (*n* = 4–5). *P < 0.05. Scale bars: 100 μm. Survival statistics for **A**–**D **calculated by Mantel Cox log rank test. Quantitative statistics for **E**–**G** calculated by *t* test.

We treated recipients of allogeneic heart grafts exposed to prolonged CIS with CTLA4Ig plus either anti-TNF mAb or isotype control to test whether TNF plays a role in graft rejection in the CIS model. We observed that, while recipients treated with CTLA4Ig alone rejected their grafts with a median survival time (MST) of 42 days, addition of anti-TNF mAb (Figure 1A) prolonged graft survival to MST > 100 days (*P* < 0.05). We then examined whether RIPK1-dependent cell death participates in the rejection of grafts exposed to prolonged CIS using necrostatin-1, a small molecule that inhibits the kinase activity of RIPK1 previously shown by others to prevent programmed cell death (51). Peritransplant administration of necrostatin-1 prolonged allograft survival compared with vehicle-treated recipients (Figure 1B). To confirm the observation obtained using the pharmacological approach, we utilized B6 *Ripk1^D138N^* donor heart allografts that specifically lack RIPK1’s kinase activity. This mutation inhibits the ability of RIPK1 to induce death (52). When we exposed these heart grafts to 8 hours of CIS and transplanted them into CTLA4Ig-treated WT BALB/c recipients (Figure 1C), we observed significantly prolonged allograft survival compared with similarly treated congenic WT control heart allografts (MST > 90 days, *P* < 0.01).

To further delineate the cell death pathway involved, we examined the survival of grafts deficient in downstream death-signaling molecules RIPK3 or MLKL, noting that direct interrogation of CASPASE 8–dependent cell death (required for apoptosis) is not possible due to embryonic lethality (53). We exposed B6 *Ripk3^–/–^* and B6 *Mlkl^–/–^* hearts to 8 hours of CIS and transplanted them into CTLA4Ig-treated BALB/c recipients (Figure 1C). These experiments confirmed, in our model system, previous reports that donor *Ripk3* deficiency prolongs heart graft survival versus WT donors (43) and showed that rejection kinetics of these RIPK3-deficient hearts was not different from that of *Ripk1^D138N^* donors (*P* = 0.84, Figure 1C). Donor *Mlkl* deficiency also resulted in prolonged allograft survival (*P* = 0.19 versus *Ripk3* deficiency), together providing additional support for the conclusion that RIPK1-dependent necroptosis plays a significant role in cardiac allograft rejection (49, 50).

To further test the notion that RIPK1-dependent cell death critically participates in allograft rejection, we performed complementary experiments using donor grafts with a gain-of-function mutation. All genetic manipulations that result in a gain of function in RIPK1-dependent death are embryonic lethal, with the exception of mice with a defect in SHARPIN. The *cpdm* mouse strain has a spontaneous loss-of-function mutation in the *Sharpin* gene that results in defective M1-linked ubiquitination of RIPK1 and induces spontaneous multiorgan inflammation (54). This functional *Sharpin* deficiency augments programmed cell death (Supplemental Figure 1), which is completely reversible by inactivating the kinase function of RIPK1 (19). To determine the impact of increased donor heart RIPK1-dependent cell death on graft survival, we exposed B6 *Sharpin^cpdm^* hearts to 8 hours of CIS and transplanted them into CTLA4Ig-treated BALB/c recipients (Figure 1D). These studies showed significantly accelerated rejection kinetics versus WT allograft controls (MST of 14 days, *P* < 0.05). Histological examination of the rejected heart allografts showed mononuclear infiltrates typical of acute cellular rejection, without evidence of spontaneous graft necrosis (data not shown). When we treated a separate set of *Sharpin^cpdm^* heart allograft recipients with anti-TNF mAb (or isotype control), we observed prolonged graft survival beyond that of untreated WT controls (Figure 1D), providing additional evidence linking T cell–mediated heart allograft rejection to TNF-initiated, RIPK1-dependent programmed cell death.

IHC analyses of heart grafts obtained from additional groups of mice harvested 7 days after transplant showed significantly less TUNEL staining in the *Ripk1^D138N^* allografts versus WT controls (Figure 1E) and that anti-TNF treatment diminished intragraft cell death in both WT and *Sharpin^cpdm^* (Figure 1, F and G), further connecting TNF production to activation of cell death pathways in the allograft.

**Figure 2:**
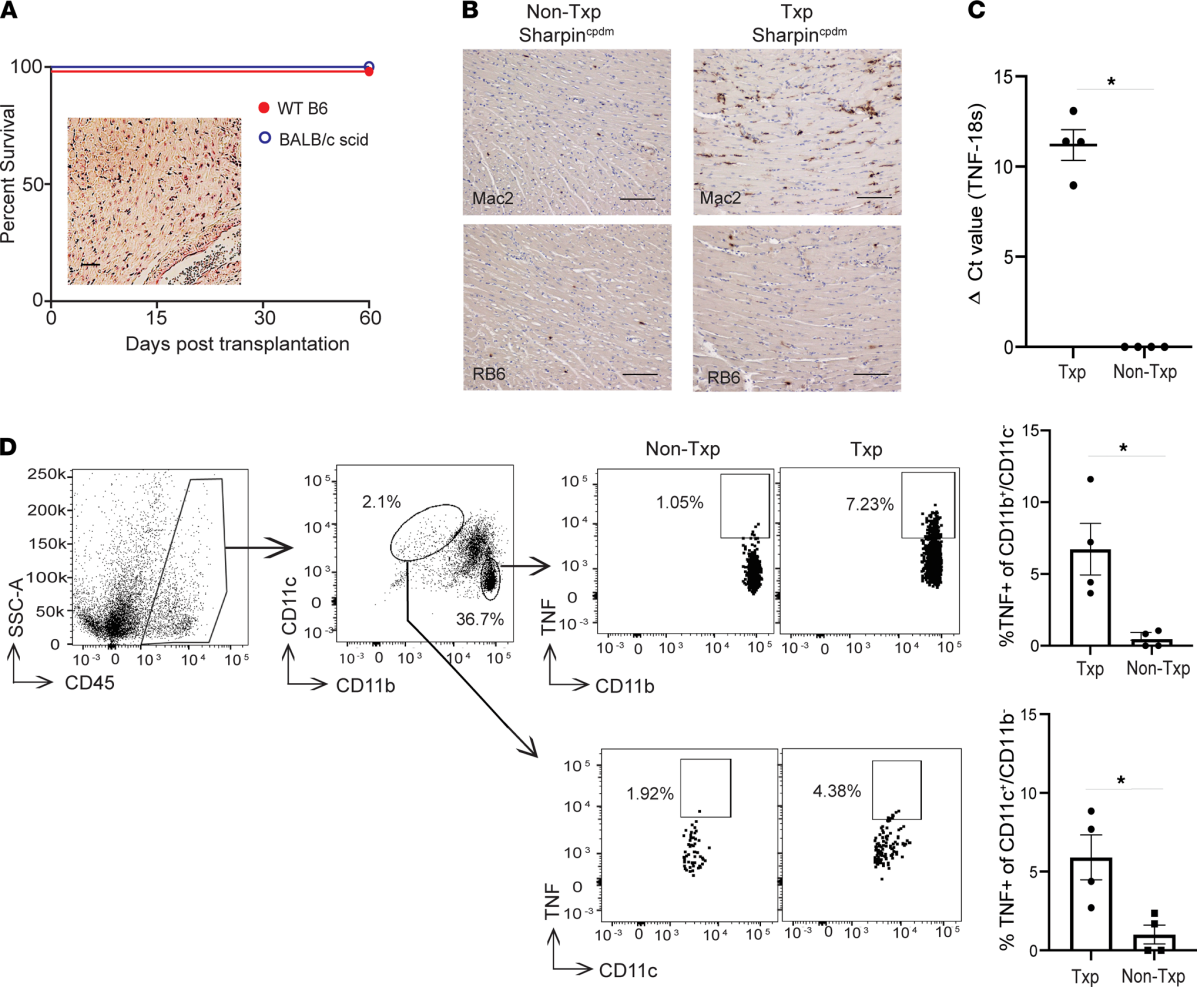
SHARPIN-deficient allografts survive indefinitely in syngeneic and immunodeficient recipients. (**A**) Survival of *Sharpin^cpdm^* allografts subjected to 8h CIS and transplanted into syngeneic WT B6 or allogeneic BALB/c scid recipients without immunosuppression (*n* = 4/group) and a representative photomicrograph (inset) of H&E-stained B6 *Sharpin^cpdm^* heart harvested from a BALB/c *scid* recipient on day 60, showing minimal mononuclear infiltration, preservation of myocyte architecture, and a normal artery (lower right). Scale bar: 25 μm. (**B**) Representative IHC showing statistically significant increased macrophage (Mac2, top) and neutrophil (RB6, bottom) staining of untransplanted *Sharpin^cpdm^* (left) and day 6 posttransplant *Sharpin^cpdm^* hearts (right) (*n* = 4/group and *P* < 0.05 for both by *t* test. Scale bar: 100 μm). (**C**) Bulk cardiac mRNA transcript qPCR ΔCt scores (TNF-18s) of naive and day 6 posttransplant *Sharpin^cpdm^* allografts. No detectable TNF gene transcript was found in hearts obtained from naive mice (*n* = 4/group, *P < 0.05 by *t* test). (**D**) Gating strategy and representative flow plots of naive (top) and day 6 posttransplant *Sharpin^cpdm^* allografts (bottom) graft infiltrating TNF-producing cells gated on the CD45^+^CD11b^+^CD11c^–^ and CD45^+^CD11b^–^CD11c^+^ populations with quantification (right) (*n* = 4/group, *P < 0.05 by *t* test).

When we next exposed *Sharpin^cpdm^* B6 hearts to 8 hours of CIS and then transplanted them into groups of either syngeneic B6 or allogeneic BALB/c *Prkdc^scid^* (*scid*) recipients (lacking T and B cells), the *Sharpin^cpdm^* grafts survived indefinitely and showed normal histology > 60 days after transplant (Figure 2A). Thus, despite enhanced sensitivity to cell death, rejection of *Sharpin^cpdm^* grafts was not due to enhanced sensitivity to cell death per se and only occurs when alloreactive T cells are present in the recipient.

TNF is established as the dominant initiator of RIPK-1–dependent cell death (10, 11, 15, 17). The indefinite survival of the *Sharpin^cpdm^* allografts in the BALB/c *scid* recipients suggested that TNF from innate cells in the BALB/c *scid* recipients is insufficient to induce cell death and rejection of *Sharpin^cpdm^* grafts. To further test for a role of innate immune cell–produced TNF (including myeloid cell production of TNF), we transplanted additional BALB/c *scid* recipients with *Sharpin^cpdm^* allografts and performed analyses on day 6 after transplant. We observed infiltration of transplanted hearts by neutrophils and macrophages (Figure 2B), upregulation of intragraft TNF gene transcript (Figure 2C) compared with naive control hearts, and production of TNF by graft infiltrating CD11b^+^/CD11c^–^ myeloid cells and CD11b^–^/CD11c^+^ DCs (Figure 2D). Together, the data support the conclusion that TNF is locally produced within the allograft by innate immune cells, and while TNF is required to activate the RIPK1-dependent cell death pathways in the allograft to cause graft rejection, it is T cell–derived TNF that mediates graft rejection in this model system.

### Kinetics of graft rejection does not correlate with frequency of alloreactive T cells.

**Figure 3:**
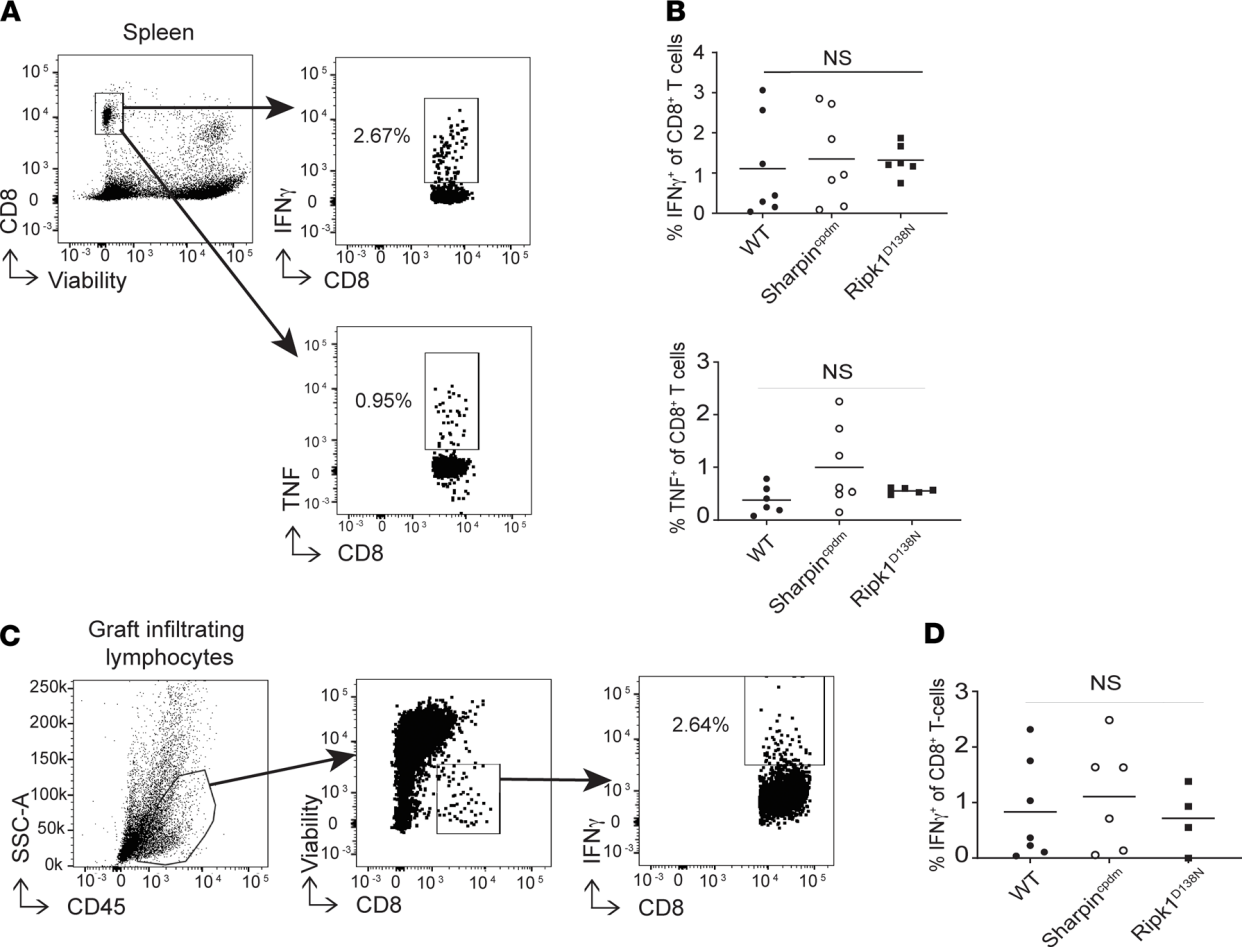
Allograft genetic sensitivity to programmed cell death does not influence frequencies of post-transplant alloreactive T cells. (**A** and **B**) Representative flow plots (**A**) and quantified frequencies (**B**) of donor-reactive IFN-γ– (top) or TNF-producing (bottom) splenic CD8^+^ T cells 10 days after transplant (*n* = 5–7); ANOVA. (**C** and **D**) Representative gating (**C**) and quantitation (**D**) of graft-infiltrating reactive IFN-γ^+^CD8^+^ T cells 48 hours after transplant (*n* = 4–6); ANOVA. For **B** and **D**, each symbol is a biological replicate.

As work by others showed that RIPK1-initiated cell death results in HMGB1 release (43) and HMGB1/TLR4 ligations could theoretically amplify T cell expansion, we tested whether the propensity to undergo cell death within the allograft is associated with alterations in the strength of the recipient donor-reactive T cell response. We transplanted sets of WT, *Sharpin^cpdm^* (predisposed to cell death) or *RIPK1^D138N^* (resistant to cell death) B6 hearts exposed to 8 hours of CIS into CTLA4Ig-treated BALB/c recipients and quantified frequencies of splenic, donor-reactive IFN-γ– and TNF-producing CD8^+^ T cells 10 days later (Figure 3, A and B). These analyses remarkably showed no differences among the groups. In our previous work (50), we showed that endogenous memory CD8^+^ T cells infiltrate allografts within 48 hours after transplant, and these memory T cells are crucial mediators of graft rejection. Building upon this observation, we tested whether early posttransplant allograft infiltration by donor-reactive T cells could explain the differences in graft survival among donor genotypes. Quantification of graft-infiltrating IFN-γ^+^ T cells 48 hours after transplant (Figure 3, C and D) also showed no differences among the groups (WT versus *Sharpin^cpdm^* versus *RIPK1^D138N^*).

### T cell–produced TNF is required for rejection.

**Figure 4:**
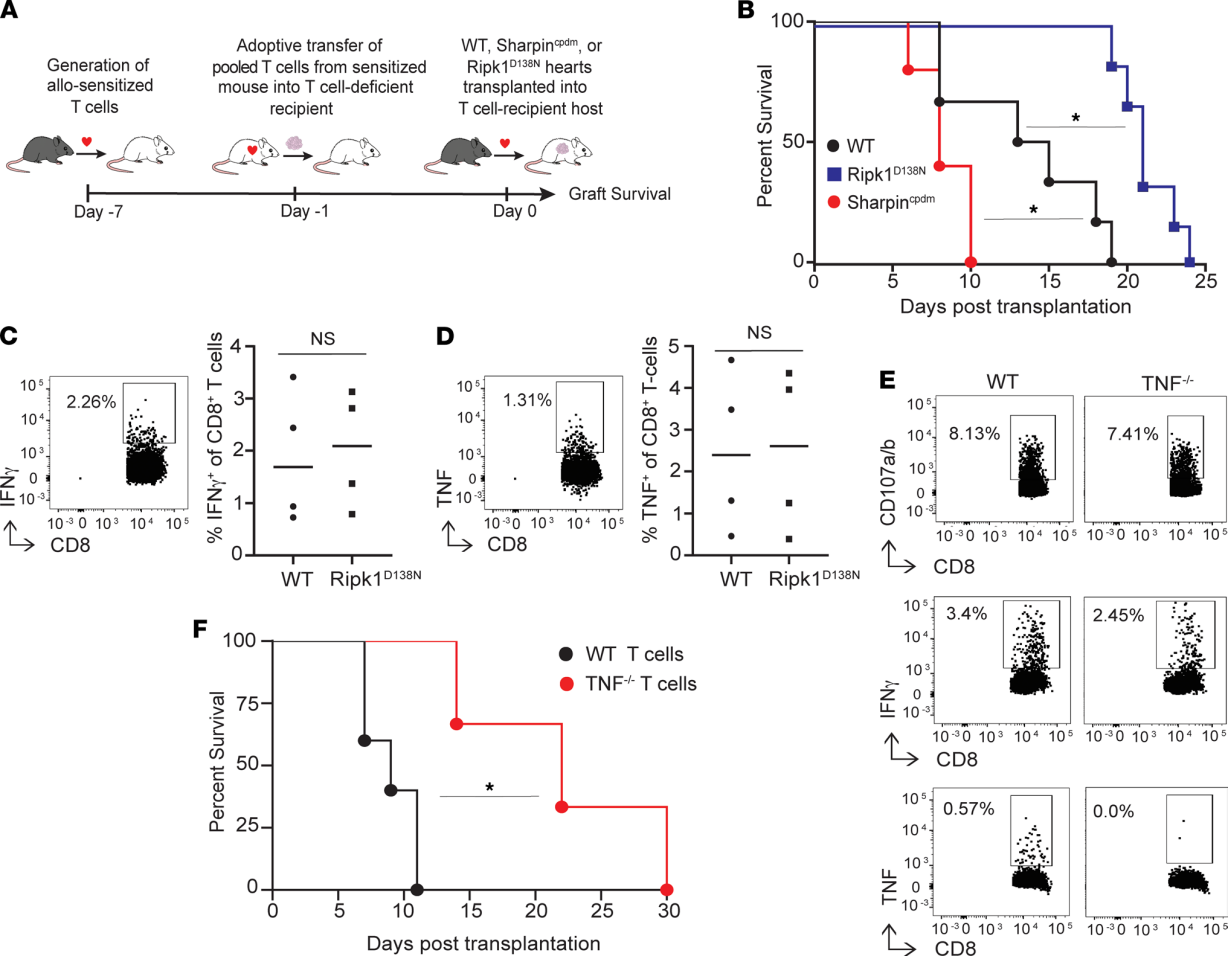
Donor graft susceptibility to RIPK1-dependent cell death determines kinetics of graft rejection. (**A**) Experimental schematic of cardiac transplant into recipients of pooled preprimed adoptively transferred T cells. (**B**) Kaplan-Meier survival curves of WT, *Sharpin^cpdm^*, or *Ripk1^D138N^* B6 hearts transplanted into BALB/c *scid* recipients of preprimed alloreactive T cells on day –1 (*n* = 6–8, *P < 0.05 versus WT condition, by Mantel Cox log rank test). (**C** and **D**) Quantified frequencies of donor reactive IFN-γ– (top) or TNF-producing (bottom) graft-infiltrating CD8^+^ T cells in *scid* recipients of adoptively transferred allo-primed T cells at 10 days after transplant (*n* = 4/group); *t* test. Each symbol is a biological replicate. (**E**) Representative (of 3 individual biological replicates) flow cytometry plots of splenic CD8^+^ T cells obtained from BALB/c–heart transplanted B6 WT or *Tnf^–/–^* recipients on day 7 showing comparable frequencies of donor-reactive CD107a/b (top), IFN-γ–producing (middle), and TNF-producing (bottom) CD8^+^ T cells. (**F**) Kaplan-Meier survival curves of WT BALB/c allografts subjected to 8h CIS and transplanted into *Rag1^–/–^* B6 recipients of preprimed WT or TNF-deficient B6 T cells (*n* = 5–6/group). *P < 0.05 relative to WT T cells by Mantel Cox log rank test.

We next formally tested the hypothesis that T cell–produced TNF is required to activate RIPK1-dependent programmed cell death within the allograft and results in rejection. We generated alloreactive effector T cells by transplanting allografts into WT BALB/c recipients without immunosuppression and isolated pooled splenic T cells from the animals on day 6 after transplant (Figure 4A). We quantified frequencies of B6-reactive IFN-γ– and TNF-producing cells (Figure 4E), and we adoptively transferred equal numbers of these anti-B6–primed T cells into syngeneic BALB/c *scid* recipients of WT, *Sharpin^cpdm^*, or *RIPK1^D138N^* B6 heart (Figure 4B). These experiments show that recipients of WT T cells rejected *Sharpin^cpdm^* grafts significantly faster (MST 8 days), yet rejected *RIPK1^D138N^* hearts significantly slower (MST 21 days), than WT grafts (MST 14 days, *P* < 0.05 relative to both other genotypes; Figure 4B). As a control to test whether the donor genotype altered T cell infiltration into the grafts, we transplanted a second set of WT or *RIPK1^D138N^* B6 heart grafts into BALB/c *scid* recipients of primed alloreactive T cells and sacrificed the recipients 10 days after transplant when all heart allografts were beating. Quantification of IFN-γ– and TNF-producing, graft-infiltrating lymphocytes (Figure 4, C and D) showed no difference between groups.

Since *Tnf^–/–^* on the BALB/c background is not available, we next generated BALB/c-reactive, B6 WT, and B6 *Tnf–/*^–^ T cells by transplanting recipients of each genotype with a BALB/c heart allograft. We isolated and pooled splenic T cells on day 6 after transplant from each genotype (Figure 4A) and normalized the frequencies of donor-reactive T cells between groups based on in vitro expression of a CTL phenotype (Figure 4E). We then adoptively transferred equal numbers of BALB/c-primed WT or *Tnf^–/–^* T cells into B6 *Rag1^–/–^* recipients of BALB/c hearts subjected to 8 hours of CIS. Graft survival analyses (Figure 4F) showed that equal numbers of *Tnf^–/–^* T cells rejected BALB/c heart allografts significantly slower than WT T cells (MST 22 days versus 9 days, *P* < 0.05). Together, the data demonstrate that T cell–derived TNF is both necessary and sufficient to mediate allograft rejection in this model system.

### Antigen-specific release of TNF by cytotoxic T cells mediates target cell cytolysis.

**Figure 5:**
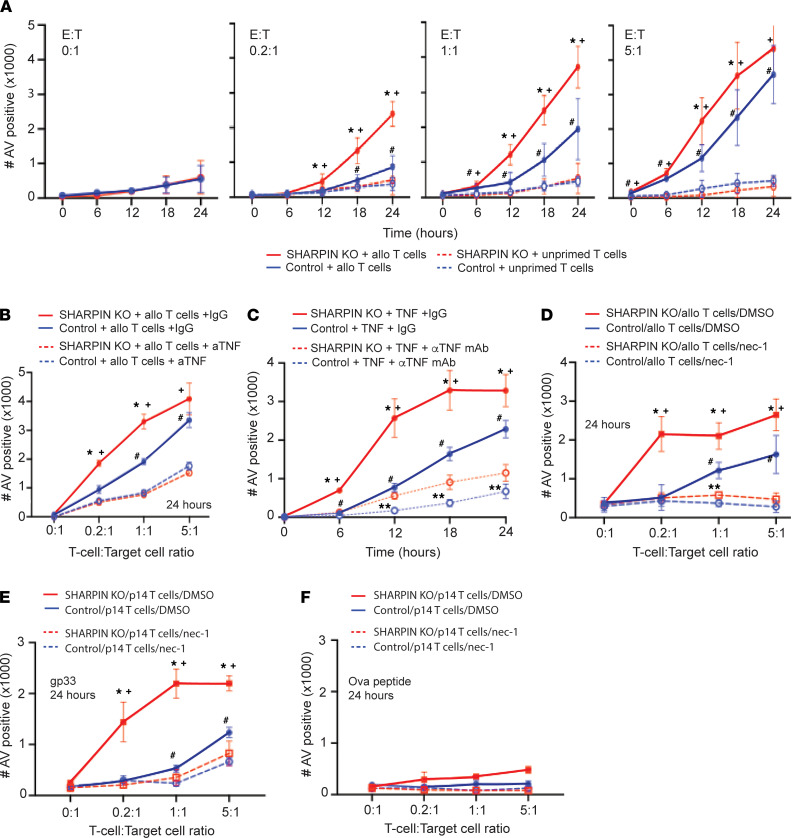
T cell–derived TNF mediates cytotoxicity via activation of target cell RIPK1–dependent cell death. (**A**) Quantified kinetics of annexin^+^ WT or SHARPIN-KO B6 MEF target cells (IncuCyte analysis) cocultured with T cells from BALB/c mice that rejected B6 hearts (solid lines) or T cells from naive BALB/c mice (dashed lines) at indicated E:T ratios. ^+^*P* < 0.05 versus SHARPIN-KO MEF + unprimed T cells; *P < 0.05 versus WT MEF + allo-primed T cells; ^#^*P* < 0.05 versus WT MEF + unprimed T cells. (**B**) Quantified killing of WT or SHARPIN-KO MEFs at 24 hours of coculture with allograft primed T cells at indicated E:T ratios ± 50 μg/mL αTNF mAb or isotype control. ^+^*P* < 0.05 versus SHARPIN-KO MEF + αTNF; *P < 0.05 versus WT MEF; ^#^*P* < 0.05 versus WT MEF + αTNF; **P < 0.05 versus SHARPIN-KO MEF + αTNF. (**C**) Quantified killing of WT or SHARPIN-KO MEFs following coculture with recombinant TNF (100 ng/mL added to all wells) ± 50 μg/mL αTNF mAb or isotype control (IgG). ^+^*P* < 0.05 versus SHARPIN-KO MEF + αTNF; *P < 0.05 versus WT MEF; ^#^*P* < 0.05 versus WT MEF + αTNF; **P < 0.05 versus SHARPIN-KO MEF + αTNF. (**D**) Quantified killing of WT or SHARPIN-KO target cells at 24 hours after coculture with allograft primed T cells (various E:T ratios) ± necrostatin-1 (nec-1) or vehicle control (DMSO). ^+^*P* < 0.05 versus SHARPIN-KO MEF + nec-1; *P < 0.05 versus WT MEF; ^#^*P* < 0.05 versus WT MEF + nec-1; **P < 0.05 versus SHARPIN-KO + nec-1. (**E** and **F**) Quantified killing of WT or SHARPIN-KO target cells at 24 hours after coculture with activated p14 TCR transgenic CD8^+^ T cells ± gp33 (**E**) or control ovalbumin (Ova) peptide (**F**) ± nec-1 or vehicle control (DMSO). For **A**–**F** each data point represents ≥ 4 technical replicates. Results are representative of at least 3 independent experiments. Statistics calculated by *t* test comparing conditions as noted.

We next directly tested the hypothesis that T cell–produced TNF mediates RIPK1-dependent cytolysis using in vitro cell killing assays. We isolated splenic, alloreactive T cells on day 6 after transplanting BALB/c recipients with B6 hearts and cocultured them in various effector/target (E:T) ratios with WT B6 murine embryonic fibroblasts (MEFs) or B6 MEFs in which we used Crispr/Cas9 to knock out SHARPIN (Supplemental Figure 2). We used an IncuCyte analyzer and annexin V staining to quantify the kinetics of target cell death. These analyses showed heightened killing of SHARPIN-KO versus WT MEFs by primed, alloreactive BALB/c T cells at all E:T ratios tested (Figure 5A). Control T cells from naive BALB/c mice plated at the same E:T ratios did not kill WT or SHARPIN-KO MEFs (Figure 5A). To test whether T cell–derived TNF, the only source of TNF within these cultures, is required for the target cell killing, we performed parallel assays in which we added a blocking anti-TNF mAb or isotype control IgG (Figure 5B). These analyses showed that TNF blockade prevented the primed, alloreactive BALB/c T cells from killing B6 target cells, regardless of SHARPIN expression. Positive control experiments confirmed that recombinant TNF directly induces greater cytotoxicity of *Sharpin*-KO versus WT MEFs and that anti-TNF mAb abrogates the effect — the latter verifying its specificity (Figure 5C). When we added necrostatin-1 to the cultures containing primed alloreactive BALB/c T cells plus WT or SHARPIN-KO MEFs (Figure 5D), we observed that the necrostatin-1 prevented killing of both sets of target cells, confirming the mechanistic role of RIPK1-dependent cell death.

Polyclonal alloreactive T cell responses involve large numbers of clones with T cell receptors (TCRs) of varying functional avidity, raising the possibility that susceptibility of target cells to TNF-initiated cell death could differentially affect T cell cytotoxic function based on expressed TCR specificity. To rule out this possibility and to test antigen specificity, we performed additional in vitro killing assays using p14 TCR transgenic CD8^+^ T cells that express TCRs with high functional avidity for the lymphocytic choriomeningitis virus–derived (LCMV-derived) gp33 epitope presented by class I MHC D^b^ (55). These assays (Figure 5E) showed more efficient killing of gp33-pulsed SHARPIN-KO versus WT MEFs by p14-reactive TCR transgenic CD8^+^ T cells, and that necrostatin-1 completely abrogated the effects. Control experiments showed no killing of OVA-peptide–loaded target cells of either genotype (Figure 5F). Together, the data indicate that T cell–derived TNF mediates antigen-specific RIPK1-dependent cytolysis of target cells; the effect occurs only during cognate interactions between the T cell and the target cells, is independent of T cell functional avidity, and is not restricted to alloreactive T cells.

### Enhanced tumor susceptibility to RIPK1-dependent death promotes T cell–derived, TNF-dependent tumor destruction.

**Figure 6:**
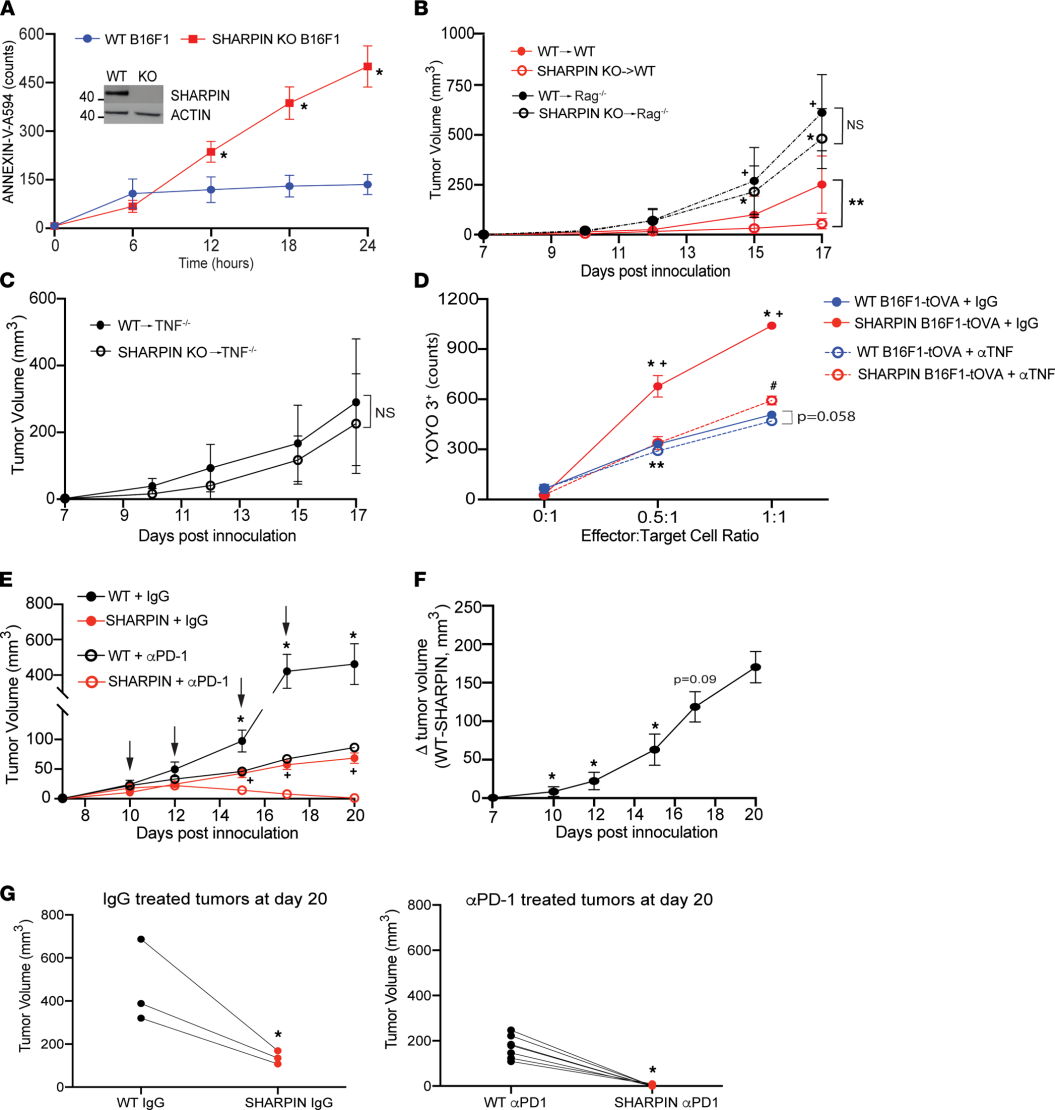
T cell–derived TNF drives RIPK1-dependent tumor killing and synergizes with checkpoint inhibition in response to murine melanoma. (**A**) Quantification of annexin^+^ WT (blue) or SHARPIN-KO (red) B16F1s cultured with TNF. *P < 0.05 (*n* = 3/group; representative of 2 independent experiments). Immunoblot of WT and SHARPIN-KO B16F1 phenotype (inset, representative of 2 independent experiments). (**B**) Volumes of WT (closed circles) or SHARPIN-KO (open circles) B16F1 tumors coinjected into WT (red) or *Rag^–/–^* (black) hosts (n=7/group). **P < 0.05 at day 17 between noted conditions; *P < 0.05 versus SHARPIN-KO→WT host; *P <0.05 versus WT→WT host). (**C**) Tumor volume kinetics of WT (closed circles) or SHARPIN-KO (open circles) B16F1 cells coinjected into *Tnf^–/–^* hosts (*n* = 7/group). (**D**) YOYO3^+^ counts of WT (blue) or SHARPIN-KO (red) B16F1-tOVA–expressing target cells cocultured with effector OT-I T cells in the presence of control IgG (square, solid line) or anti-TNF (circle, dashed line) mAb at indicated E:T ratios (*n* = 3/group). *P < 0.05 versus WT + IgG; ^+^*P* < 0.05 versus Shp + αTNF; ^#^*P* < 0.05 versus WT + αTNF; **P < 0.05 WT + IgG versus WT + αTNF). (**E**) Tumor volume kinetics of WT (black lines) or SHARPIN-KO (red lines) B16F1 coinjected into WT hosts and treated with anti-PD1 (open circles) or IgG control (closed circles). Arrows indicate days of injection. (*n* = 7 for αPD1 therapy and *n* = 3–5 for IgG control). *P < 0.05 versus WT + αPD1; ^+^*P* < 0.05 versus SHARPIN + αPD1. (**F**) Kinetic of difference in tumor size between WT and SHARPIN-KO B16F1 tumors in anti-PD1–treated hosts (*n* = 7). *P < 0.05 versus day 20. (**G**) Paired comparison of tumor size at day 20 after inoculation of IgG-treated (upper panel) or anti-PD1–treated (lower panel) hosts (*n* = 7 for αPD1 therapy and *n* = 3 for IgG control). *P < 0.05 versus WT condition. For all panels, statistics calculated between noted conditions by *t* test.

In an effort to test the generalizability of the mechanism beyond transplantation, we tested the effects of T cell–derived TNF on tumor destruction using the murine B16F1 melanoma model. We generated SHARPIN-deficient B16F1 cells using Crispr-Cas9 (Figure 6A) and confirmed that, whereas WT B16F1 cells are resistant to cell death, the SHARPIN-deficient B16F1 cells are sensitive to TNF-induced apoptosis (Figure 6A). RIPK1 is recruited to Complex II in SHARPIN-deficient B16F1 cells, providing further evidence of these cells undergo RIPK1-dependent death (Supplemental Figure 3). To test the effects of SHARPIN deficiency on tumor growth, we injected equal numbers of WT or SHARPIN-deficient B16F1 cells s.c. into opposing flanks of WT B6 hosts. While we detected WT and SHARPIN-deficient tumors in all animals within 10 days, we observed slower growth kinetics and significantly smaller SHARPIN-deficient tumors at day 17 compared with the WT control tumors (Figure 6B). When we repeated the experiments in T cell– and B cell–deficient *Rag1^–/–^* hosts, we observed no differences in the growth kinetics or sizes of the tumors by genotype (Figure 6B) and that both tumors grew faster/larger in the *Rag1^–/–^* versus WT hosts. Together with the observed differential growth in the WT animals, these results showed that an increased susceptibility to RIPK1-dependent cell death (conferred by SHARPIN deficiency) does not result in spontaneous tumor cell death, but rather sensitizes to T cell–mediated tumor destruction. To test whether TNF is required for these effects, we coinjected WT and SHARPIN-deficient B16F1 cells into *Tnf^–/–^* recipients (Figure 6C). These experiments revealed no differences in the growth kinetics/sizes of WT versus *Sharpin*-deficient tumors, indicating that lifting restraint on tumor cell sensitivity to RIPK1-dependent cell death only results in accelerated tumor destruction if both T cells and TNF are present, echoing the observations of the transplant model. Together with in vitro experiments showing the same number of OVA-specific OT-I cells more efficiently kill OVA-expressing, SHARPIN-deficient versus WT B16F1 cells via a TNF-dependent mechanism (Figure 6D), our findings demonstrate that tumor cell destruction, analogous to transplant rejection, is dependent upon T cell–produced TNF that triggers target tissue RIPK1–dependent cell death.

Since B16 melanoma cells were shown by others to be resistant to checkpoint inhibition (56, 57), we tested whether lifting restraint on programmed cell death increases the tumor’s susceptibility to anti-PD1 therapy. We again inoculated the opposing flanks of mice with WT or SHARPIN-deficient B16F1 melanomas cells, and 10 days later, when both tumors were detectable and of similar size, we initiated therapy with anti-PD1 mAb or isotype control. Consistent with previous reports, the anti-PD1 partially reduced WT tumor size/growth (Figure 6E) but remarkably, and within the same animals, eliminated the SHARPIN-deficient tumors (Figure 6, E–G), demonstrating synergism. Kinetic analysis of the difference in tumor size between the WT and SHARPIN-deficient melanomas in the mice treated with anti-PD1 showed progressive divergence within the same animals (Figure 6F), indicating a local synergy between T cell checkpoint blockade and activation of target cell programmed cell death by T cell–derived TNF.

## Discussion

T lymphocytes are established producers of TNF (47, 48), and the inability of T cells to produce TNF is one characteristic of T cell dysfunction/exhaustion (58, 59); however, the functions and associated mechanisms of T cell–derived TNF have remained elusive. Herein, we newly demonstrate that (a) T cell–derived TNF is both necessary and sufficient for optimal T cell cytotoxic function, (b) the TNF is released by antigen specific T cells during cognate interactions with their targets, (c) systemic and non–T cell–derived TNF does not participate, and (d) mechanistically, the locally released TNF functions by specifically activating target cell RIPK1–dependent cell death programs. The uncovered mechanisms have translational potential, as we showed that T cell–derived TNF contributes to heart transplant rejection and synergizes with checkpoint blockade to result in clearance of an otherwise resistant murine cancer.

The findings provide additional mechanistic insight to explain previously reported observations by others attempting to link T cell–derived TNF to clearance of select pathogens (25, 60) and to cytolysis of allogeneic endothelial cells — findings that were attributed in part to TNF driven amplification of T cell IFN-γ and CD107a/b degranulation (45, 61, 62). Our data implicate T cell–derived TNF as initiating RIPK1-dependent cell death programs within the target cells to account for the observations.

Our results add to the understanding of programmed cell death pathways in transplantation. Beyond confirming a role for RIPK3 in organ rejection (43–45), we showed that the absence of donor graft RIPK1 kinase activity prolonged allograft survival and diminished intragraft cell death, whereas the absence of functional SHARPIN in an allograft accelerated allograft rejection and enhanced intragraft cell death. As absence of allograft RIPK1 kinase activity (required for both necroptosis and apoptosis) and absence of allograft MLKL (the terminal effector of necroptosis) similarly extended graft survival, our data provide further evidence beyond the published findings using RIPK3-deficient grafts (43) that necroptosis contributes significantly to the TNF-initiated graft failure in this system. One caveat is, because we cannot test effects of donor caspase 8 deficiency (animals are embryonic lethal; ref. 53), we cannot formally rule out an additional role for apoptosis here.

Our detection of equivalent frequencies of donor-reactive, cytokine-producing splenic and intragraft T cells in mice rejecting WT and *Sharpin^cpdm^* allografts, and the indefinite survival of B6 *Sharpin^cpdm^* allografts in *scid* recipients that contain a full complement of nonlymphoid immune cells that can produce TNF, support our assertion that T cell–derived TNF and target organ cell susceptibility to TNF-initiated programmed cell death determine the kinetics of allograft survival. In previous work by others, prolonged survival of murine *Ripk3^–/–^* kidney or heart allografts was attributed in part to diminished inflammation associated with less HMGB1 production (43). Our results prompt a reinterpretation of these observed effects: the absence of donor RIPK3 in these studies increases resilience of the graft to T cell cytotoxicity dependent upon T cell derived TNF.

In clinical transplantation, our results raise the possibility that targeting cell death pathways in the graft (e.g., the use of RIPK1 kinase inhibitors), or potentially targeting TNF production, could limit T cell CTL function and improve graft outcomes without the immunosuppressive infectious complications associated with nonspecific T cell–targeted therapy. The results of an ongoing randomized controlled NIH-funded study of anti-TNF mAb (infliximab) in kidney transplant recipients, Clinical Trials in Organ Transplantation–19 (CTOT-19; clinicaltrials.gov, NCT02495077) will be highly informative in this regard. Our studies also suggest that duration of graft survival could be a function of the genetics and epigenetics of the donor organ. We previously proposed that reduced expression of antideath genes in the graft (analogous to the *Sharpin* defect) could lead to accelerated rejection, whereas reduced expression of prodeath genes (analogous to the *Ripk1* kinase defect) could lead to prolonged graft survival (63). These effects on susceptibility to RIPK1-dependent cell death on graft survival are distinct from previous work showing that necroptosis but not apoptosis principally mediates ischemia reperfusion injury in native or transplanted organs (29, 30, 64). Nonetheless, as ischemia reperfusion injury can negatively influence transplant outcomes (65, 66), together, the data indicate that allograft durability could potentially be predicted by analyzing the relative expression levels of prosurvival versus prodeath signaling molecules.

While TNF was discovered in the search for an effector mechanism to account for the antitumor effects of endotoxin (5), it was abandoned as a primary antitumor therapy except in the context of isolated limb perfusion in combination with melphalan (67). Our data provide a plausible explanation for why systemic administration of TNF failed as an effective therapy for malignancies: TNF must be delivered by tumor-reactive T cells during cognate interactions with the tumor targets in order to activate RIPK1-dependent cell death and tumor destruction.

Despite the documented failure of systemically administered TNF as an antitumor therapy, continued interest remains in exploring TNF in this context (68, 69). Ongoing work implicates programmed cell death pathways as part of a biologically relevant process in controlling malignancy (39, 70). Together with the recognition that effective tumor immunotherapy relies in part on overcoming T cell exhaustion (characterized in part by absence of TNF production) to augment the antitumoral T cell response (71), these observations support approaches aimed at augmenting T cell–derived TNF production in antitumor immunity. Indeed, tumor infiltrating chimeric antigen receptor (CAR) T cells engineered to be resistant to exhaustion were shown to have increased TNF production (among other markers of polyfunctionality) compared with WT controls, and this was associated with improved per-cell cytotoxicity and better in vivo control of established tumors in mice (72). In further support, several recent Crispr-Cas screens in tumor cells have identified TNF death–signaling molecules to be critical in controlling tumor growth during immune checkpoint blockade (69, 73, 74). Genetic loss of TNF and its death-signaling molecules (e.g., *Tnfr1* and *Casp8*) led to uncontrolled tumor growth, whereas loss of molecules involved in ubiquitinating RIPK1 (e.g., *Traf2* and *Birc2/3*) resulted in tumor rejection following immune checkpoint blockade (69, 73–75). Similar to what we proposed for allograft survival (63), the level of pro– and anti–programmed cell death molecules within the tumor may determine response to immunotherapy that includes TNF-producing T cells. A recent study identified activation of the TNF/NF-κB cell survival pathway in tumor cells resistant to checkpoint blockade and CD8^+^ T cell–mediated killing (75), highlighting the central role of this pathway in immune escape by cancer cells. The data from our studies provide a mechanistic explanation for these previous findings and further support that enhancing tumor-reactive T cell–derived TNF (perhaps one consequence of overcoming T cell exhaustion) and sensitizing tumor cells to programmed cell death may be useful strategies in tumor immunotherapy.

Beyond transplantation and tumor immunology, this newly identified function of T cell–derived TNF to enhance T cell cytotoxicity via activation of RIPK1-dependent cell death likely evolved in response to infectious pathogens. Multiple animal and human studies implicate programmed cell death as a key mediator of intracellular pathogen clearance (76–78), and TNF (in part produced by T cells) is also required for optimal control of listeria monocytogenes (LM) (60) and mycobacterium tuberculosis (25). These previous publications suggested that T cell–produced TNF is required to clear high-dose LM infections and control pathogens in the latter phase of mycobacterium tuberculosis infections (requiring robust T cell responses). We speculate that the protective effects of the T cell–produced TNF were due in part to triggering of programmed cell death pathways within the target cells and to subsequent diminished resilience of the infected cells to T cell cytotoxicity.

The required deactivation of the early ubiquitin-dependent cell death checkpoint to permit RIPK1’s association with death-signaling molecules also allows for selective activation of cell death in target cells. In fact, the virulence factor YopJ encoded by yersinia pestis, a bacteria that requires the kinase activity of RIPK1 for clearance, is one such RIPK1 switch (79, 80). Exploiting a pathogen-derived component to permit TNF-mediated programmed cell death in an infected cell provides discrimination such that only the infected cell succumbs to TNF-mediated killing, while neighboring noninfected cells are spared and may in fact respond to TNF by expressing NF-κB–dependent inflammatory mediators. The nature of the pathogen-derived switch to turn on RIPK1 death signaling in other pathogens remains to be uncovered. Within the context of graft rejection in our transplant model, it is currently unclear what may be acting as the switch to result in death/rejection of WT donors in the absence of infection. Potentially as we previously speculated (63), a shift in the balance over time between pro– and anti–programmed cell death molecules in the WT donor grafts could lead to the switch.

Altogether, our studies reveal a previously unrecognized mechanism through which T cell–derived TNF facilitates antigen-specific T cell cytotoxicity by activating RIPK1-dependent cell death within target cells. Our findings provide the foundation for future work to assess applicability of the mechanisms in preclinical models of other immune-mediated processes including autoimmunity, response to infectious pathogens, and malignancy and to assess whether manipulating target cell resilience to cytotoxicity can improve outcomes in people with T cell–mediated diseases, including transplant rejection and cancer.

## Methods

### Study design.

This study was designed to explore the biologic function of T cell–derived TNF and its interactions with tissue RIPK1-dependent cell death. We utilized heterotopic murine cardiac transplant models and the B16F1 melanoma tumor system, along with in vitro cell culture experiments that allowed us to specifically manipulate target tissue sensitivity to programmed cell death and/or T cell production of TNF. Data collection for in vivo experimental animals was terminated early if the mice reached prespecified IACUC humane end points. Outliers were predefined as diverging more than 2 SDs from the mean. No outliers were excluded. Analysis of the data was done in an unblinded manner.

### Animals and procedures.

C57BL/6 (*H-2^b^*), *Tnf^–/–^* (*H-2^b^*) (81), and BALB/c (*H-2^d^*) mice were purchased from the Jackson Laboratory or bred from our in-house colony. CBySmn.CB12-*Prkdc^scid^*/J (*scid*) and B6.129S7-*Rag1^tm1Mom^*/J (*Rag1^–/–^*) were purchased from the Jackson Laboratory. C57BL/KaLawRij-*Sharpin^cpdm^*/RijSunJ was provided by John Sundberg (The Jackson Laboratory). *Ripk3^–/–^* strain was provided by Vishva Dixit (Genentech) (82). *Ripk1^D138N^* strain was provided by Michelle Kelliher (University of Massachusetts, Worcester, Massachusetts, USA) and Manolis Pasparakis (University of Cologne, Cologne, Germany) (52). *Mlkl^–/–^* strain was provided by Warren Alexander (WEHI, Melbourne, Australia) (83). p14 TCRtg mice were originally obtained on a B6.CD90.1 background from M. Oldstone (Scripps Research, La Jolla, California, USA). CD8^+^T cells from these mice (p14 cells) are specific for the dominant LCMV-GP_33–41,_ gp33, determinant restricted by D^b^). All animals were maintained in specific pathogen–free conditions. Experiments were performed using littermates or with mice maintained in the same room and/or were cohoused within the same cages to limit potential effects of microbiome differences.

Heterotopic heart transplants were performed as previously described by our laboratories (84). Allografts were subjected to prolonged (8-hour) CIS with preservation in ice-cold sterile University of Wisconsin solution. Recipient mice received 250 μg CTLA4Ig (Orencia, Bristol Myers Squibb) i.p. on the day of surgery when indicated. For experiments testing TNF blockade, recipients were additionally given 250 μg anti-TNF Ab (BioXCell, clone XT3.11, catalog BE0058) or control anti–horseradish IgG (BioXCell, clone HRPN, catalog BE0088) by i.p. injection on day 0 only for WT allografts and serially every 5 days for 2 weeks after transplant in recipients of SHARPIN-deficient grafts.

We performed 2–4 prolonged CIS transplants per day based on animal and surgeon availability. The transplant survival data shown in Figures 1 and 2 represent cumulative results of multiple surgery experiments for each group of animals, performed over a 9-month period as one large experiment. All grafts that failed within 72 hours after transplant were considered technical failures (< 10% of transplants). We constructed one survival curve for all of the WT controls obtained over the course of the study. As noted in the figure legend for Figure 1, we split the survival data into 2 separate graphs (Figure 1, C and D) for ease of visualization. We included the same survival curve for control WT transplants in each of these graphs.

### Tumor experiments.

In total, 1 × 10^5^ WT or *Sharpin*-KO (generation described below) B16F1 melanoma cells were injected s.c. into opposing flanks of WT, *Tnf^–/–^*, or *Rag1^–/–^* B6 mice. Tumor volume was measured 3 times/week starting 7 days after inoculation. For checkpoint blockade experiments, 200 μg anti-PD1 (RMP1-14, BioXCell, catalog BE0146) or isotype control (rat IgG2a, BioXCell, catalog BE0089) were injected i.p. 3 times/week starting on day 10 after inoculation. Only mice that had detectable WT and *Sharpin*-KO tumors at day 10 were included in the therapeutic experiments.

### Flow cytometry.

Viability dye was purchased from Invitrogen (catalog 65-0865-14). The following mAbs were purchased from Invitrogen: CD107a (ebio1D4B, catalog 50-1071-82), CD107b (ebioABL-93, catalog 50-1072-82), Isotype IgG2aκ (eBR2a, catalog 50-4321-80), IFN-γ (XMG1.2, catalog 45-7311-82), TNF (MP6-XT22, catalog 12-7321-82), CD11b (M1/70, catalog 45-0112-82), and CD11c (N418, catalog 11-0114-85). From Tonbo, we purchased CD4 (GK1.5, catalog 60-0041-U100). From BioLegend, we purchased CD8 (53-6.7, catalog 100725). From eBioscience, we purchased CD45 (30-F11, catalog 48-0451-82).

Intracellular cytokine staining was performed as previously described (84). Briefly, live splenocytes from experimental mice were cocultured 1:1 with freshly isolated antigen presenting cells (APCs; negative fraction of EasySep Mouse 90.2 positive isolation kit II, Stemcell Technologies, catalog 18951) from naive donor mice at 37°C overnight. After 18 hours of incubation, Golgiplug (BD Biosciences, catalog 555029) was added to the culture wells for 4 hours. After surface staining, cells were subsequently fixed/permeabilized (eBioscience, catalog 00-5523-00) and stained for intracellular cytokines per the manufacturer protocols.

### PCR.

RNA was isolated from allograft cardiac tissue by using TRIzol (Thermo Fisher Scientific) and synthesized into cDNA per manufacturer’s protocol. cDNA was reverse transcribed using the High Capacity cDNA Reverse Transcription Kit (Thermo Fisher Scientific, catalog 4368814) as per the manufacturer instructions. Quantitative PCR (qPCR) was performed with TaqMan primers 18s (Thermo Fisher Scientific, Mm03928990_g1) and TNF (Thermo Fisher Scientific, Mm00443258_m1) and run on the CFX96 Real-Time System (Bio-Rad Laboratories).

### Adoptive transfer of WT alloreactive T cells into SCID recipients.

We sensitized WT BALB/c recipients with transplanted WT B6 donor hearts. Six days later, we processed recipient spleens and isolated total T cells by negative magnetic bead separation (EasySep Mouse Tcell Isolation kit, Stemcell Technologies, catalog 19851A); 12 × 10^6^ T cells were injected retro-orbitally into SCID mice. We cultured a portion of the isolated T cells overnight at 37°C with B6 APCs (negative fraction of EasySep Mouse 90.2 positive isolation kit II, Stemcell Technologies, catalog 18951) to confirm alloreactivity (IFN-γ and TNF production) by flow cytometry as described above. The following day, we transplanted the adoptive hosts with heart allografts from WT, *Sharpin^cpdm^*, or *RIPK1^D138N^* mice after 8 hours of CIS. We determined graft failure by cessation of a palpable heartbeat and confirmed by visual inspection/histological analysis.

### Adoptive transfer of Tnf^–/–^ T cells.

We transplanted WT BALB/c hearts into WT or *Tnf^–/–^* B6 recipients and, 6 days later, isolated splenic T cells as above. We cultured a portion of the isolated T cells with BALB/c APCs and anti-CD107a/b flow cytometry antibodies for 30 minutes at 37°C to assess degranulation by flow cytometry and allow for real-time normalization of alloreactivity. We cocultured a separate fraction of isolated T cells with allogeneic APCs overnight to confirm alloreactivity by cytokine production the following day as above. We injected (retro-orbital) 5 × 10^6^ WT T cells or an equal number of alloreactive *Tnf^–/–^* T cells (normalized using the degranulation assay) into *Rag1^–/–^* mice on the day of T cell isolation.

### Generation of Sharpin-KO MEF and B16F1 cell lines.

We seeded 5 × 10^5^ WT (H-2^b^) MEF cells (85) or B16F1 melanoma cells (provided by Miriam Merad, Icahn School of Medicine at Mount Sinai, New York, USA) into each well of a 6-well plate in complete media. We replaced the medium with 1 mL of Opti-MEM. After complexing 2 ng of control or *Sharpin*-targeting plasmid with 10 μL of Lipofectamine2000 in 300 μL of Opti-MEM for 5 minutes, we dispensed the solution dropwise to the designated well of the 6-well plate. We replaced the culture medium the next day. Two days after transfection, we replaced the medium again with 2 mL of complete media containing 5 μg/mL of puromycin plus plasmid PX459 V2.0 encoding a control nontargeting sgRNA (5′-GCGAGGTATTCGGCTCCGCG-3′) or targeting *Sharpin* (5′-CGTGCACTTCCTCGACCCCG-3′) (Genscript). We confirmed absence of SHARPIN by immunoblotting with anti-SHARPIN (Proteintech, catalog 14626-1-AP), and bulk puromycin-resistant cells were used in experiments to minimize founder effects.

### Cell death quantification.

We seeded target cells at 5 × 10^3^ cells/well in 96-well tissue culture plates. Eighteen to 24 hours after plating, we replaced the culture media with 100 μL of phenol red–free media containing effector cells, treatments, and fluorescently labeled recombinant annexin V (86) or 500 nM of the cell-impermeable viability dye YOYO3. Cells were treated as indicated and immediately subjected to SPARKL real-time cell death analysis using an IncuCyte ZOOM (Essen Biosciences) (87). The processing definitions for the IncuCyte ZOOM were as follows. Red (AF594–annexin V): parameter, TopHat; radius, 30 μm; threshold, 5.0; edge sensitivity, –34; Area, >100 μm. Red (YOYO3): parameter, TopHat; radius, 25 μm; threshold, 2.0; edge sensitivity, –45; area, >150 μm. Data shown are the mean of triplicate samples ± SD and are representative of at least 3 replicated experiments.

### The p14 cell cytotoxicity assay.

We pulsed target cells seeded as above with 10 μM gp33 (synthesized at > 98% purity, GenScript Biotech) or control ova peptide (MilliporeSigma, A5503) for 2 hours on the day of the experiment and then washed them to remove excess peptide. We activated total splenic T cells isolated from naive p14 T cell receptor transgenic mice (EasySep Mouse T cell Isolation kit, Stemcell Technologies, catalog 19851) using anti-CD3/CD28 dynabeads (Thermo Fisher Scientific, 11452D) for 4 hours. After washing, we added the p14 cells to the target cells with or without necrostatin-1 (Thermo Fisher Scientific, catalog 4800655MG; 30 μM final concentration) or an equal volume of DMSO control, and we quantified annexin V staining as outlined above.

### B16F1 cell cytotoxicity assay.

A truncated OVA (tOVA) encoding residues 212–386 was fused to GFP with a nuclear localization signal and cloned into pBABE-Hygro (Addgene, 1765) (88). Retrovirus generated using this plasmid was transduced into control and *Sharpin*-KO B16F1 prior to hygromycin selection. T cells from transgenic OT-1 mice were purified using the EasySep Mouse T cell isolation kit and cultured for 24 hours in wells coated with anti-CD3 and -CD28 to activate T cells. On the day of the experiment, target B16F1 cells were cocultured with activated OT-1 T cells with anti-TNF (50 μg/mL) mAb or IgG control. YOYO3 positivity was quantified as outlined above.

### Coimmunoprecipitation.

For FADD immunoprecipitation, 2 confluent plates of B16F1 cells per sample were stimulated and then lysed in buffer containing 20 mM Tris (pH 7.4), 150 mM sodium chloride, 10% glycerol, 0.2% NP-40, 0.1 mM sodium orthovandate, 5 mM β-glycerophosphate and Protease Inhibitor Cocktail Set V for 20 min on ice. Lysates were cleared by centrifugation at 10,000*g* at 4°C, and protein concentration was measured using Pierce BCA (Thermo Fisher Scientific). An equivalent amount of protein in each sample was immunoprecipitated by rotating with 1 μg of FADD mAb clone G4 (Santa Cruz Biotechnology, catalog sc-271748) overnight at 4°C. Immune complexes were precipitated with Protein A/G beads. After extensive washing, the beads were eluted with SDS-sample buffer at 70°C for 20 minutes. Sequential blotting with anti-RIPK1 mAb clone D94C12 (Cell Signaling Technology, 3493S) and anti-FADD mAb clone EPR5030 (Abcam, ab124812) was carried out. In all, 50 μg aliquots of total lysates were also resolved by SDS-PAGE and sequentially blotted with antibodies against RIPK1 (clone D94C12, Cell Signaling Technology, 3493S), FADD (clone EPR5030, Abcam, ab124812), SHARPIN (Proteintech, 14626-1-AP), and β-actin (clone 8H10D10, Cell Signaling Technology, 3700S).

### IHC and TUNEL staining.

We detected apoptotic cells in paraffin embedded tissue sections with a TUNEL Kit (Abcam, catalog ab206386) following the protocol provided by the manufacturer, using Diaminobenzidene chromogen and counterstained with Methyl Green. We purchased Mac2 antibody (clone M3/38, Cedarlane, catalog CL8942AP) and RB6 antibody (clone RB6-8C5, BioXCell, catalog BE0075) and used them per manufacturer’s instructions.

### Statistics.

We performed statistical analysis using Prism GraphPad software version 8. We calculated survival statistics by Mantel Cox log rank test. For all other experiments, we used 2-tailed Student’s *t* test with normal distribution, with adjustments for small samples sizes as required, or 2-way ANOVA when comparing experimental multiple groups. Quantitative data shown with mean ± SEM where relevant. *P* < 0.05 was considered significant.

### Study approvals.

All mouse studies were approved by the IACUC at Icahn School of Medicine at Mount Sinai.

## Author contributions

PSH and ATT provided funding for the work (multiple-private investigator grant) and together conceptualized the project; oversaw the work, including guiding design, implementation, and interpretation of all experiments; and wrote and edited the manuscript. NC contributed to analysis of experiments shown in Figures 1 and 2; designed, analyzed, and interpreted experiments in Figures 3–6; prepared all figures; wrote the initial draft of the manuscript; and edited the manuscript. RLA contributed to initial conceptualization of the project, performed the transplant experiments shown in Figures 1 and 2, and performed pilot studies supporting experiments shown in Figure 3. MC generated the cells used and performed some of the experiments in Figure 5, expanded the scope of the work to tumor models by performing and analyzing experiments shown in Supplemental Figures 2 and 3 and in Figure 6, and edited the manuscript. WMB analyzed the pathology of the transplant experiments, performed and analyzed IHC and TUNEL staining of graft tissue, and edited the manuscript. RLF helped to design the TNF blocking experiments, provided reagents (Figure 4), and edited the manuscript. JKH provided technical assistance for experiments shown in Figure 3 and edited the manuscript. JDG and JEC oversaw and assisted in the analysis of experiments done on the IncuCyte analyzer shown in Figures 5 and 6 and edited the manuscript. MAK provided KO animals for the work, provided consultative support, and edited the manuscript. VIP and YL performed all heart transplant procedures and edited the manuscript. DH provided p14 mice, assisted in the design and implementation of experiments shown in Figures 5 and 6, and edited the manuscript. The order of co–senior authors was decided by mutual agreement of the senior authors.

## Supplementary Material

Supplemental data

## Version 1 11/09/2021

In-Press Preview

## Version 2 12/22/2021

Electronic publication
