# Supplementary material for: T cell–derived tumor necrosis factor induces cytotoxicity by activating RIPK1-dependent target cell death
Source: JCI Insight. 2021 Dec 22;6(24):e148643. doi: 10.1172/jci.insight.148643 (PMC8783689; doi:10.1172/jci.insight.148643)
Supplement: Supplemental data [file jciinsight-6-148643-s096.pdf]

Supplemental Figures:

Supplemental Figure 1

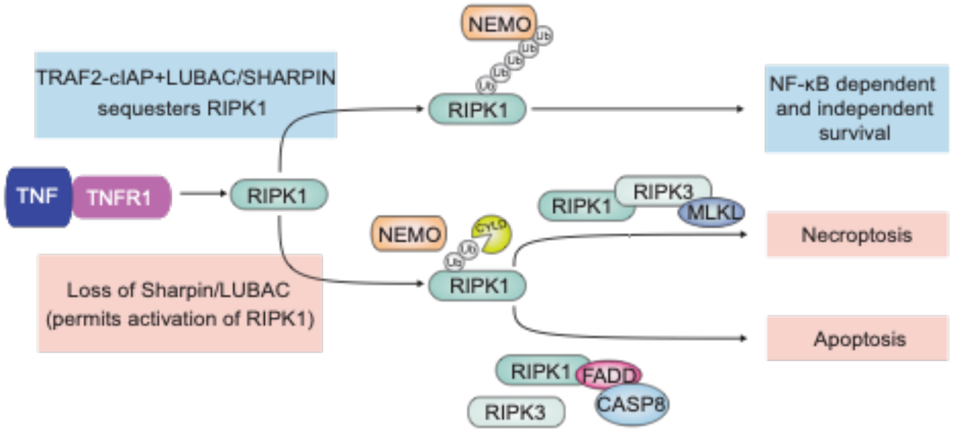

Supplemental Fig 1: Schematic overview of TNF/TNFR induced signaling pathways. Ubiquitination of RIPK1 catalyzed by TRAF2/cIAP1/2 and the LUBAC (containing SHARPIN) E3 ligases to conjugate lysine 63 (K63) and linear (M1) poly-ubiquitination, respectively, stabilizes the RIPK1's interaction with NEMO, a component of the IKK complex. RIPK1-NEMO association inhibits RIPK1 from interacting with the death-signaling Complex II containing FADD, CASPASE 8 and RIPK3, and permits the phosphorylation of RIPK1 by IKK family kinases. In the absence/dysfunction of the E3 ligases and/or the upregulation of deubiquitinases, RIPK1 interacts with FADD and CASPASE 8 to initiate downstream activation of CASPASE 8 and trigger apoptosis. RIPK3 is a non-essential enhancer of apoptosis via this pathway (1, 2). If CASPASE 8 activity is inhibited, deubiquitinated RIPK1 induces activation of RIPK3 and ultimately activates mixed lineage kinase domain-like protein (MLKL)-dependent necroptosis.

Supplemental Figure 2

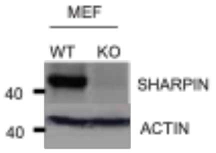

Supplemental Figure 2: Immunoblot of WT or SHARPIN knockout (SHARPIN KO) murine embryonic fibroblasts (representative of 2 independent experiments).

Supplemental Figure 3

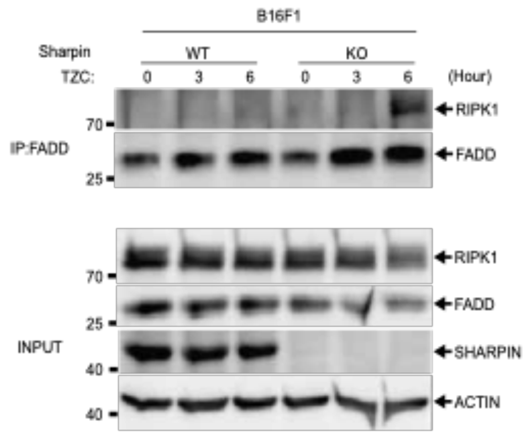

Supplemental Figure 3. Activation of apoptosis pathway and recruitment of RIPK1 to DISC in SHARPIN KO but not WT B16F1 cells. B16F1 cells of both genotypes were stimulated with TNF (100 ng/ml) in the presence of zVAD-FMK (20 μM) and cycloheximide (1 μg/ml) for 0, 3 and 6 hours. Lysates from these cells were immunoprecipitated with anti-FADD to isolate the death-signaling Complex II, followed by western blotting with anti-RIPK1. Prior to immunoprecipitations, aliquots of the total lysates were reserved (input) and analyzed by western blotting with the indicated antibodies.

References:

1. Dondelinger Y, Aguileta MA, Goossens V, Dubuisson C, Grootjans S, Dejardin E, et al. RIPK3 contributes to TNFR1-mediated RIPK1 kinase-dependent apoptosis in conditions of cIAP1/2 depletion or TAK1 kinase inhibition. *Cell Death Differ.* 2013;20(10):1381-92.
2. Lalaoui N, Boyden SE, Oda H, Wood GM, Stone DL, Chau D, et al. Mutations that prevent caspase cleavag of RIPK1 cause autoinflammatory disease. *Nature.* 2020;577(7788):103-8.
